# Supplementary material for: Analyzing technology acceptance and perception of privacy in ambient assisted living for using sensor-based technologies
Source: PLoS One. 2022 Jul 5;17(7):e0269642. doi: 10.1371/journal.pone.0269642 (PMC9255774; doi:10.1371/journal.pone.0269642)
Supplement: Attachment — Submitted filename: Response to reviewers.docx [file pone.0269642.s002.docx]

**Dear Editor, dear Reviewers,**

Thank you very much for your valuable comments and the opportunity to revise our paper entitled “Analyzing technology acceptance and perception of privacy in ambient assisted living for using sensor-based technologies”. We are very thankful for your and the reviewers’ suggestions which lead to significant improvements of our manuscript. We addressed all comments in our best possible way and provided our responses within a table (for a better overview) indicating in which parts of the manuscript the corresponding changes are located. Please, find our responses in the Table below.

| **Editor/**  **Reviewer #** | Reviewer Issue/Comment to Be Addressed | Response/Action Taken | **Document Location** |
| --- | --- | --- | --- |
| Reviewer 1 | “The paper describes an interesting and fascinating aspect of the adoption of wearable device – the lifelogging - to support mobility and autonomy of people with disabilities. The exploratory study was focus on general population to investigated the users’ technology acceptance and the privacy perception. Results of the 312 participants showed that the privacy is considered the main aspect that involve the acceptance of the assistive technology.” | Thank you very much for your review and your valuable suggestions. We have included accordingly the particular modifications into our manuscript as reported below. | --- |
| Reviewer 1 | “P7 line 163: I invite the authors to describe the definitions of PEU and PU.“ | Thank you for this great suggestion. We have now added the definitions as suggested. | Technology acceptance of sensor-based lifelogging technologies  Lines 160–165 |
| Reviewer 1 | “It is not clear how the authors present the devices to participants to be evaluated. Do they use at home as trial? Do they receive a short presentation? I invite the authors to add these information.” | Thank you for this comment. We actually have explicitly described how our participants were confronted with the concrete technology (lines 270–281), but for a better understanding we still supplemented information in the text and we now added pictures of the sensors that we presented to the participants embedded in a schematic structure overview of the online survey (Fig 2). | Data collection  Lines 270ff and 299–300 |
| Reviewer 2 | “The manuscript reports interesting aspects on technology acceptance and privacy perceptions in private environment of German adults. Moderate revisions are necessary to improve the value of the manuscript.” | We are thankful for your review and your valuable advices. We now included some modifications and hope that our work can soon publicly contribute to a more sustainable research and user-centered development of lifelogging technologies. | --- |
| Reviewer 2 | “The abstract has to report the name of the survey and technology adopted briefly.” | Thank you for this great suggestion. We complimented the missing information and we additionally paraphrased the abstract so that it is now clearer for the reader. | Abstract |
| Reviewer 2 | “A more concise Introduction should implement the reading from the Journal audience. It is too long and you may not focus on the main topics.” | Thanks for the recommendation. We now shortened the Introduction section leaving there only the main topics and the logical consequences for the users of the investigated technology. | Introduction |
| Reviewer 2 | “Some typos are also present: please see for instance line 257, May instead of “Mai”. Check carefully the whole ms for that.” | We reviewed the whole manuscript more precisely for typo and syntax errors. | Whole manuscript,  References |
| Reviewer 2 | “Finally, I suggest to summarize survey parts into a Table in order to allow readers a fast knowledge and reduce the length of the report.” | Thank you for this suggestion. In accordance with the another reviewer’s comment, we now added a Figure (Fig 2), which presents a schematic structure of the online survey. While this has—unfortunately—not reduced the length of the manuscript at this point, it has substantially improved the comprehensibility and overview of our method. | Method  Data collection  Lines 299–300 |
| Editor / Journal requirements | “Please state what role the funders took in the study.” | Thank you for this advice. We added your suggested statement “the funders had no role in study design, data collection and analysis, decision to publish, or preparation of the manuscript.” to the financial disclosure. | Funding Statement |
| Editor / Journal requirements | “In your Data Availability statement, you have not specified where the minimal data set underlying the results described in your manuscript can be found. PLOS defines a study's minimal data set as the underlying data used to reach the conclusions drawn in the manuscript and any additional data required to replicate the reported study findings in their entirety. All PLOS journals require that the minimal data set be made fully available” | Thank you very much for this comment and information. We prepared a data sheet referring to the results in our manuscript and uploaded it as “supporting information”. | Data Availability Statement |
| Editor / Journal requirements | “Please include your full ethics statement in the ‘Methods’ section of your manuscript file. In your statement, please include the full name of the IRB or ethics committee who approved or waived your study, as well as whether or not you obtained informed written or verbal consent. If consent was waived for your study, please include this information in your statement as well.” | Thank you for this valuable advice. We added a full ethic statement at the beginning of the methods section. | Methods (Ethic Statement) |
| Editor / Journal requirements | “Please review your reference list to ensure that it is complete and correct. If you have cited papers that have been retracted, please include the rationale for doing so in the manuscript text, or remove these references and replace them with relevant current references. Any changes to the reference list should be mentioned in the rebuttal letter that accompanies your revised manuscript. If you need to cite a retracted article, indicate the article’s retracted status in the References list and also include a citation and full reference for the retraction notice.” | Thank you for this information. In line with a thorough proofreading, we reviewed our reference list and confirm the references to be complete and correct. | References |
